# Supplementary material for: UV Light Causes Structural Changes in Microplastics Exposed in Bio-Solids
Source: Polymers (Basel). 2023 Nov 4;15(21):4322. doi: 10.3390/polym15214322 (PMC10647429; doi:10.3390/polym15214322)
Supplement: Supplementary file 1 [file polymers-15-04322-s001.zip › polymers-2622523-supplementary.pdf]

Supplementary Materials

UV Light Causes Structural Changes in Microplastics Exposed in Bio-Solids

Somayye Sadat Alavian Petroody [1], Seyed Hossein Hashemi [1], Luka Škrlep [2], Branka Mušič [2], Cornelis A.M. van Gestel [3] and Andrijana Sever Škapin[2,4,*]

(a)

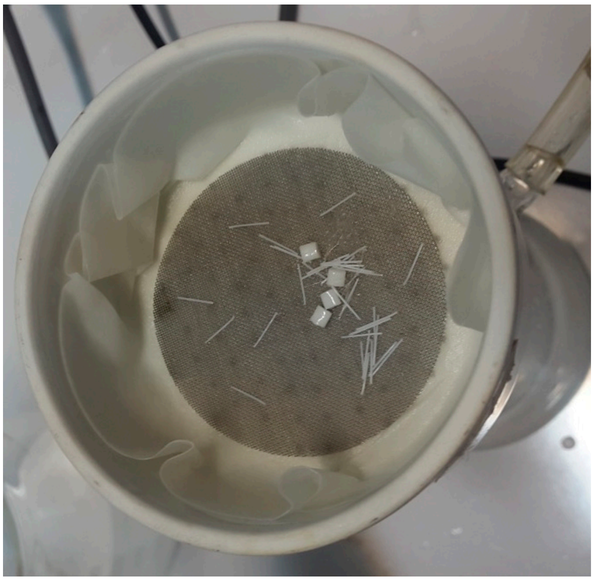

(b)

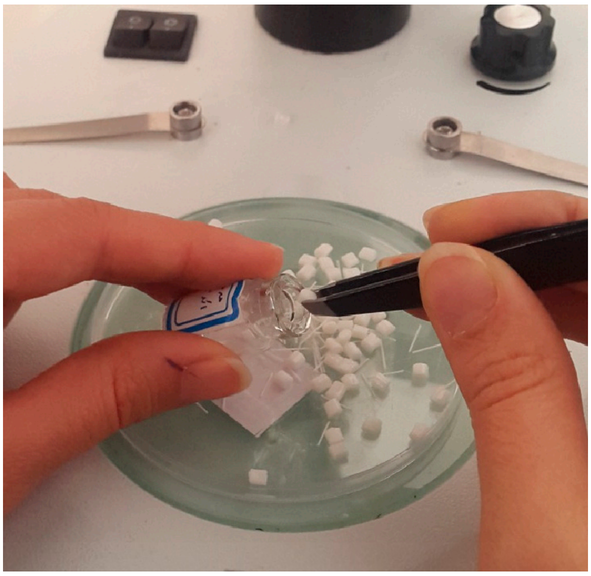

(c)

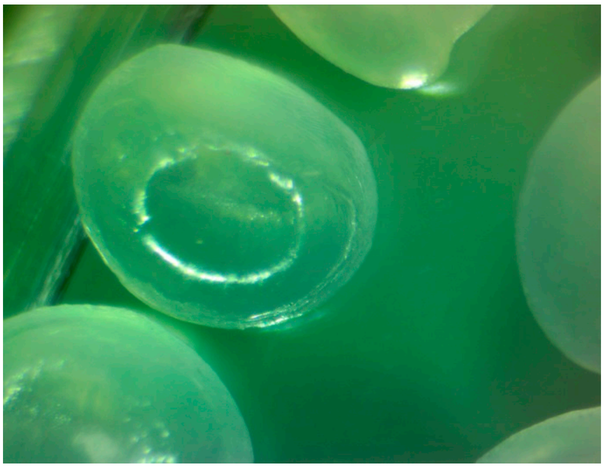

Figure S1. Photos of the samples: (a) lines and granules on filter system, (b) granules, (c) zoom of granules.

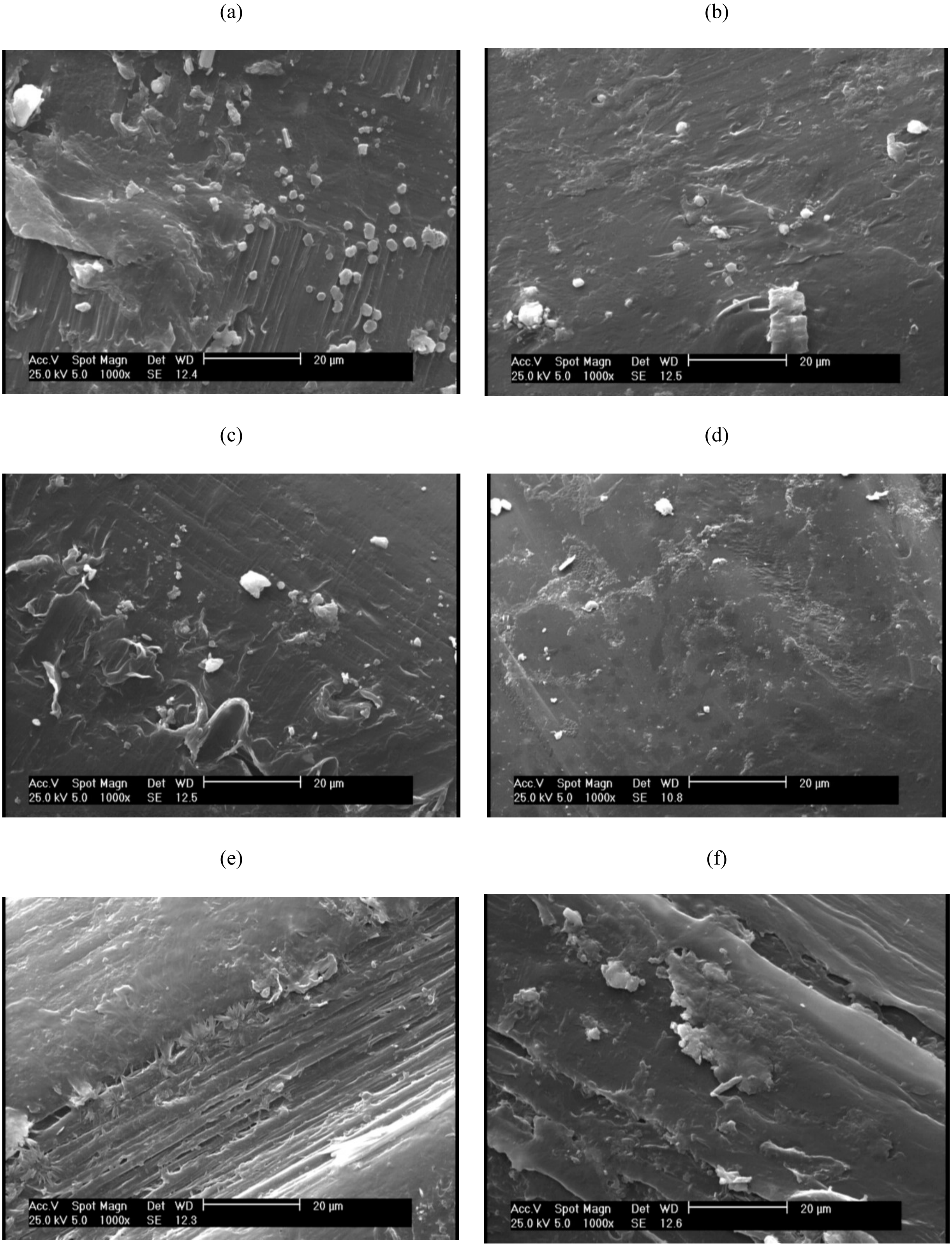

Figure S2. SEM images of: - **first line** (a) PE lines and (b) PP lines after 48h exposure to UVA; **second line** (c) PE granules and (d) PP granules after 24h exposure to UVA; **third line** (e) PE granules and (f) PP granules after 48 h exposure to UVC.

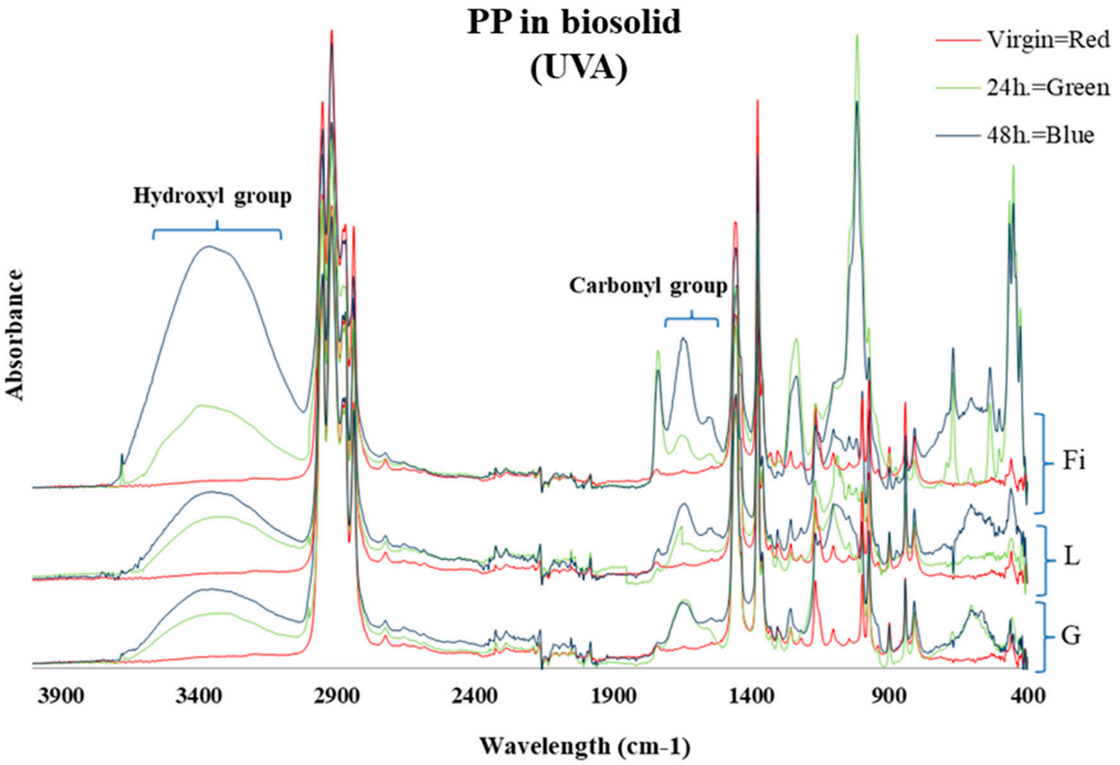
PP in biosolid
(UVA)
Virgin=Red
24h.=Green
48h.=Blue
Hydroxyl group
Carbonyl group
Absorbance
Fi
L
G
3900
3400
2900
2400
1900
1400
900
400
Wavelength (cm-1)

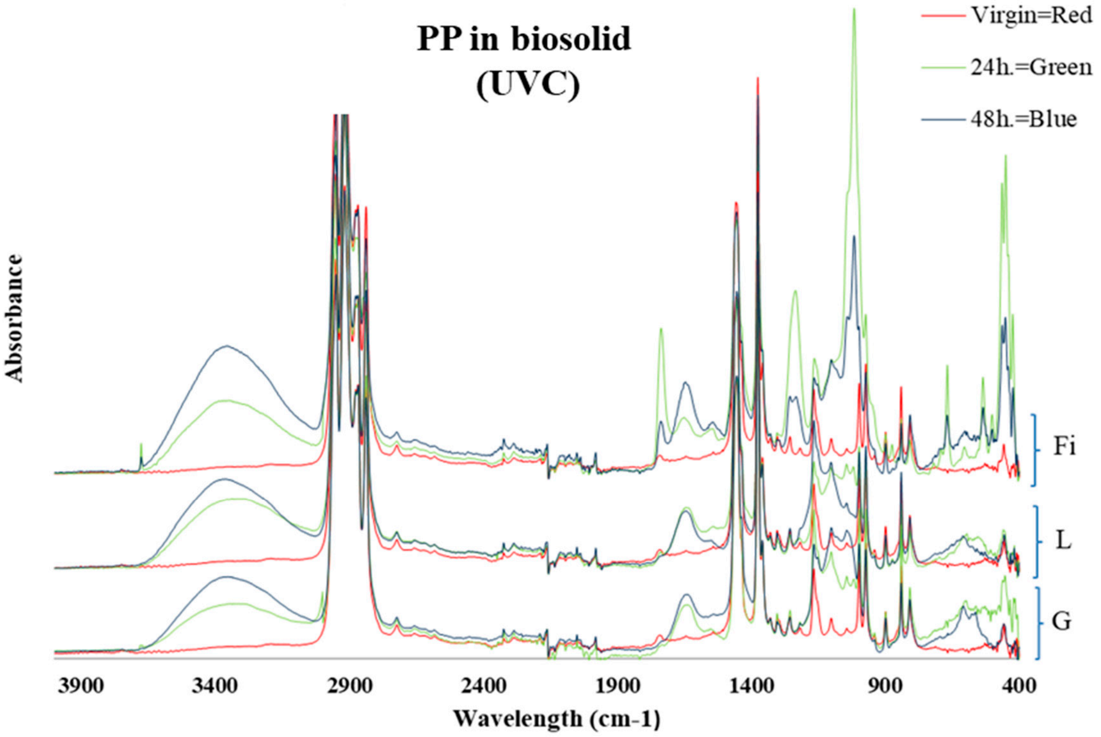
PP in biosolid
(UVC)
Virgin=Red
24h.=Green
48h.=Blue
Absorbance
Fi
L
G
3900
3400
2900
2400
1900
1400
900
400
Wavelength (cm-1)

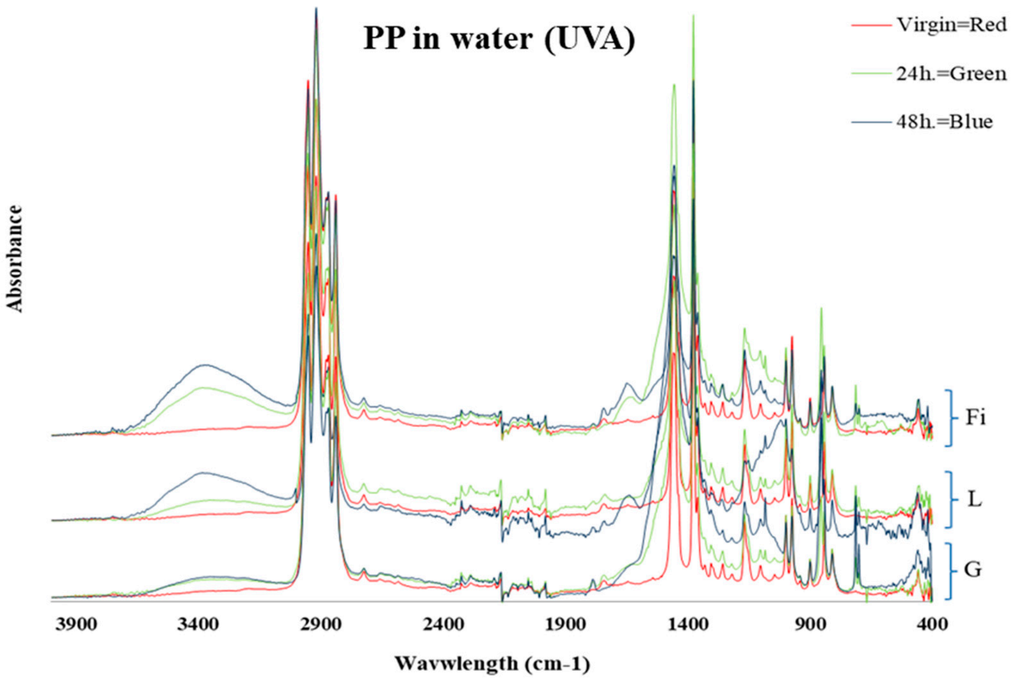
PP in water (UVA)
Virgin=Red
24h.=Green
48h.=Blue
Absorbance
Fi
L
G
3900
3400
2900
2400
1900
1400
900
400
Wavwlength (cm-1)

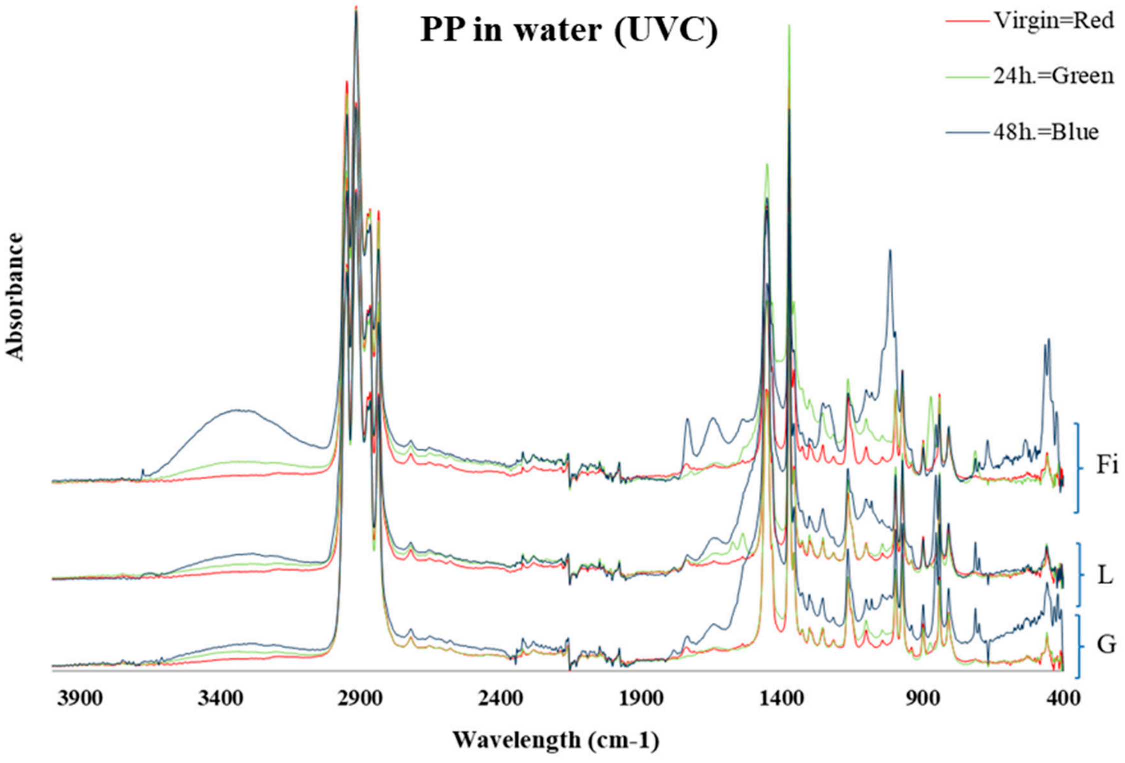
PP in water (UVC)
Virgin=Red
24h.=Green
48h.=Blue
Absorbance
Fi
L
G
3900
3400
2900
2400
1900
1400
900
400
Wavelength (cm-1)

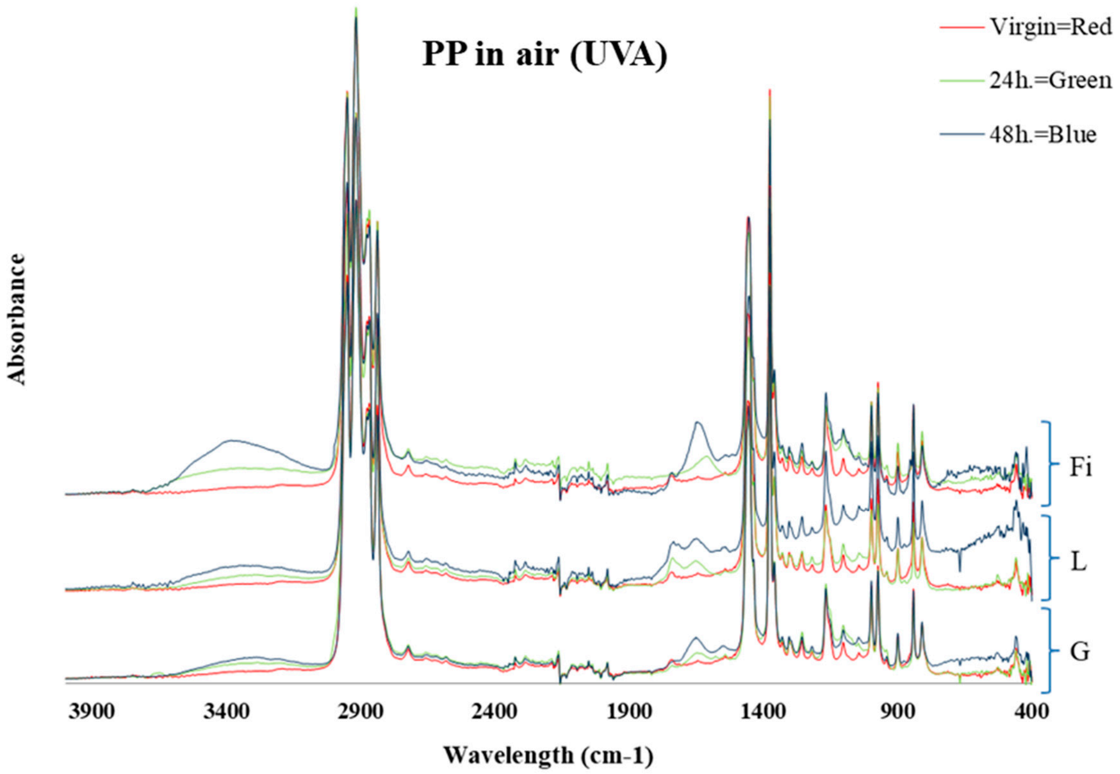
PP in air (UVA)
Virgin=Red
24h.=Green
48h.=Blue
Absorbance
Fi
L
G
3900
3400
2900
2400
1900
1400
900
400
Wavelength (cm-1)

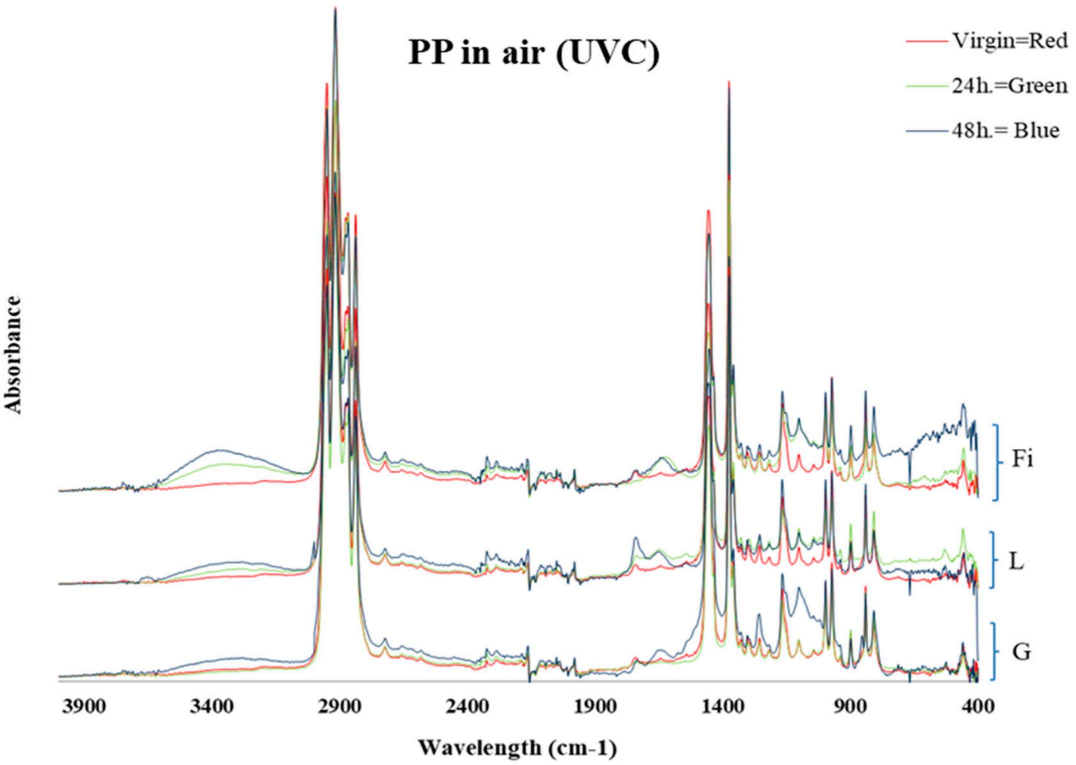
PP in air (UVC)
Virgin=Red
24h.=Green
48h.= Blue
Absorbance
Fi
L
G
3900
3400
2900
2400
1900
1400
900
400
Wavelength (cm-1)

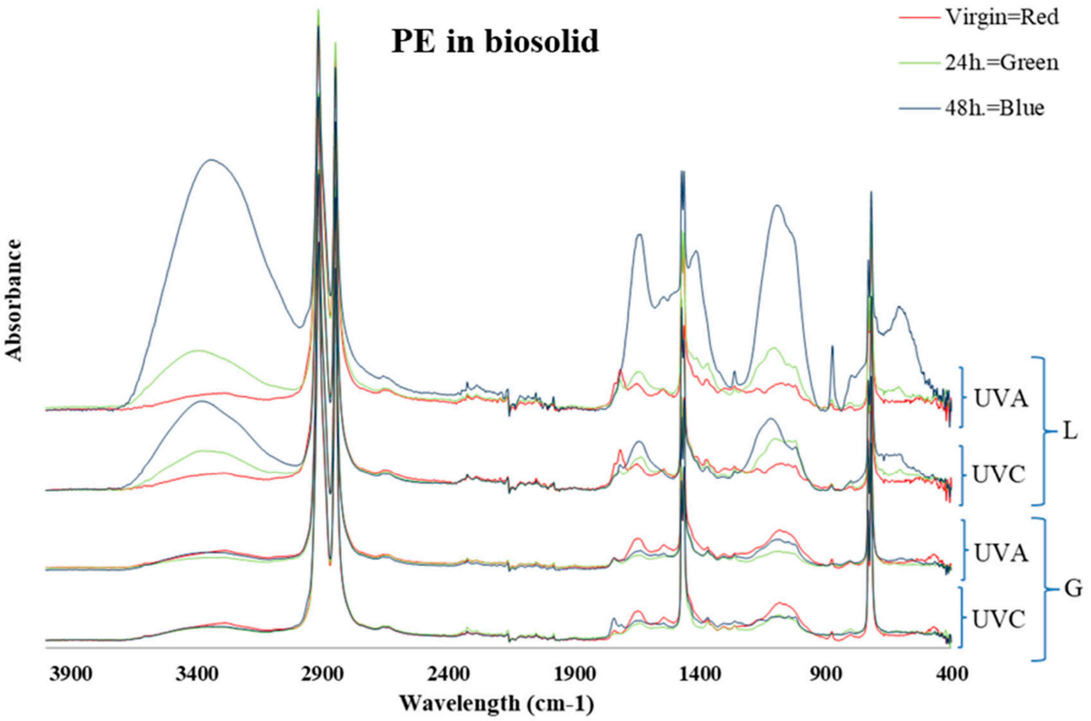
PE in biosolid
Virgin=Red
24h.=Green
48h.=Blue
Absorbance
UVA
UVC
UVA
UVC
L
G
3900
3400
2900
2400
1900
1400
900
400
Wavelength (cm-1)

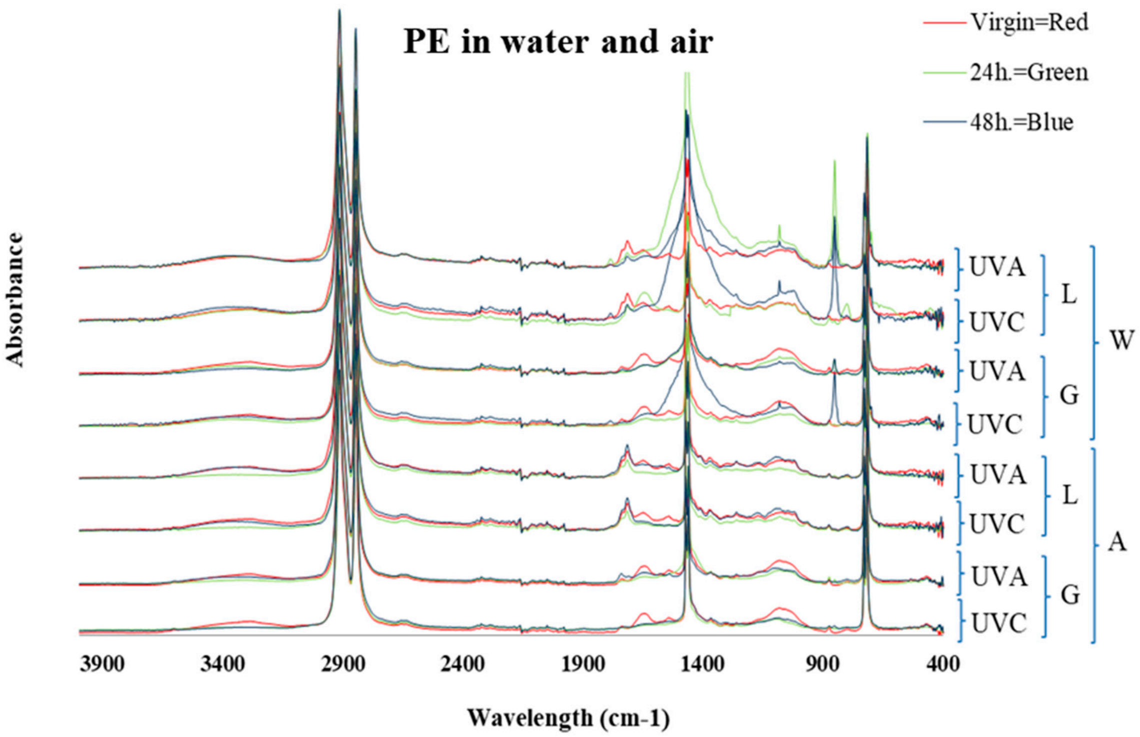
PE in water and air
Virgin=Red
24h.=Green
48h.=Blue
Absorbance
UVA
UVC
UVA
UVC
UVA
UVC
UVA
UVC
L
G
L
G
W
A
3900
3400
2900
2400
1900
1400
900
400
Wavelength (cm-1)

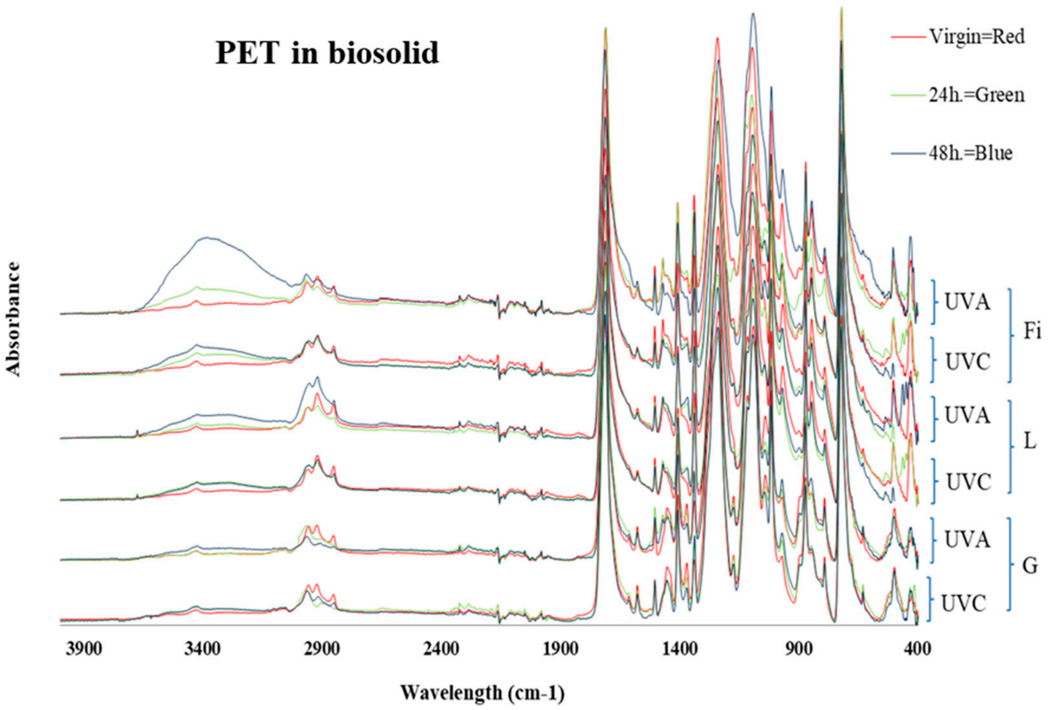

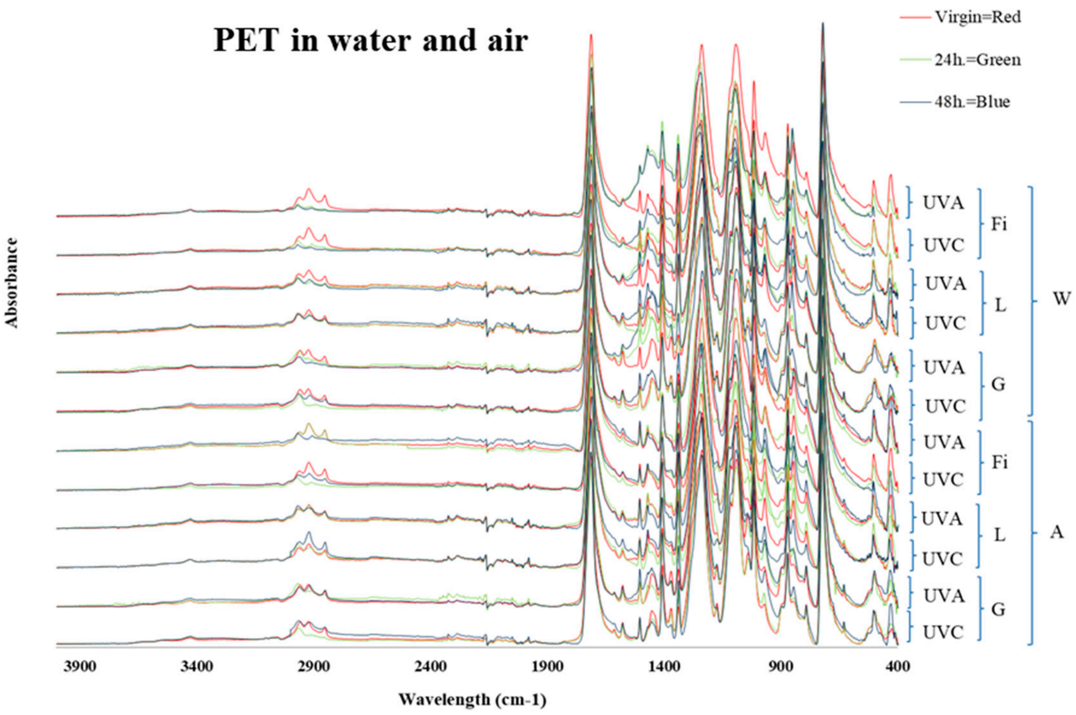

Figure S3. FTIR spectra (400-4000 cm^{-1}) and induction of carbonyl (C=O; ~1700 cm^{-1}) and hydroxyl (OH, ~3300 cm^{-1}) groups in different shapes of polypropylene (PP) microplastics in air, water and biosolids exposed for 24 h and 48 h to UV-A and UV-C light, at 70 °C, and compared with virgin (reference) samples. (Y axis is related to the adsorption peaks). Fi: Filament, L: line, G: Granule, W: water, A: Air.

Table S1. CI and HI for virgin samples (the samples at 0 min of UV irradiation).

HI	PP	PE	PET
Fi	1.04	-----	0.98
L	1.04	1.02	0.99
G	1.04	1.01	1

CI	PP	PE	PET
Fi	1.03	----	1
L	1.03	1.03	1
G	1.03	1.03	1
